# Comparative physiological and immunological impacts of *Moringa oleifera* leaf and seed water supplements on African catfish (*Clarias gariepinus*): effects on disease resistance and health parameters

**DOI:** 10.1186/s12917-025-04717-9

**Published:** 2025-05-07

**Authors:** Howyda G. Gaber, Nehal A. Younis, Sohair Y. Saleh

**Affiliations:** 1 Department of Physiology, Faculty of Veterinary Medicine, Cairo University, Giza, PO 11221 Egypt; 2 Department of Aquatic Animal Medicine and Management, Faculty of Veterinary Medicine, Cairo University, Giza, PO 11221 Egypt

**Keywords:** Natural immunostimulant, Antioxidant capacity, Cytokine expression, Liver enzymes, Bacterial challenge

## Abstract

This study evaluated the differential effects of *Moringa oleifera* leaf and seed powders (0.8 g/L) as water supplements on the physiological and immunological responses of African catfish (*Clarias gariepinus*) over six weeks. Leaf supplementation enhanced growth performance (final weight: 120.5 ± 0.7 g & gain% 65.2) and disease resistance, while seed supplementation elicited adverse physiological outcomes. Fish treated with seed powder exhibited reduced growth performance (gain % 11.2), elevated stress markers (glucose: 113.3 ± 3.8 mg/dL; cortisol: 27.4 ± 1.3 μg/dL), and compromised liver function (ALT: 30.2 ± 1.1 U/L; AST: 53.0 ± 1.6 U/L), evident through histological changes. Both treatments modulated immune responses, significantly upregulating pro-inflammatory serum cytokines (*TNF-α*, *IL- 1β*, IL- 6) and increasing gene expression in kidney and spleen tissues, with seeds group showing more pronounced elevations (*TNF-α*: 4.15-fold, *IL- 1β*: 3.15-fold in spleen) compared to moderate increases in leaves group (*TNF-α*: 2.48-fold, *IL- 1β*: 1.62-fold). Oxidant/antioxidant analysis revealed contrasting effects: leaf treatment enhanced superoxide dismutase activity (SOD) and reduced lipid peroxidation, malondialdehyde (MDA), while seed treatment compromised SOD defense and increased oxidative biomarker (MDA). Upon challenge with *Aeromonas hydrophila*, leaf-treated fish maintained 100% survival, while seed-treated and control groups showed 85% and 60% survival rates, respectively. These findings underscore the potential of *M. oleifera* leaf powder as an effective water supplement for enhancing growth and disease resistance in aquaculture, while cautioning against the use of seed powder due to its adverse physiological effects.

## Introduction

The African catfish (*Clarias gariepinus*) stands as one of the most economically significant freshwater aquaculture species globally [27]. Widely cultivated across Africa, Asia, and parts of Europe, African catfish plays a vital role in food security and livelihood generation, particularly in developing countries [17]. In Egypt, the aquaculture sector has experienced remarkable growth in recent decades, with *C. gariepinus* emerging as a key species due to its rapid growth rate [25]. However, despite its potential, African catfish production in Egypt has fluctuated, with data from the General Fisheries Authority for Resources Development showing a decline from 13,622 tons in 2012 to 8,475 tons in 2019 [29]. As intensive aquaculture practices expand to meet rising global protein demands, effective fish health management has become critical for sustainable production [15]. In this context, natural feed additives have garnered increasing attention as sustainable alternatives to synthetic supplements, offering potential benefits for enhancing fish health and disease resistance [2].

Among these natural additives, *Moringa oleifera *Lam., commonly known as the "miracle tree" or "drumstick tree," has gained significant interest in aquaculture due to its multifaceted benefits [30]. Native to northern India and Pakistan, *M. oleifera*is now widely cultivated in tropical and subtropical regions [55]. Its leaves are particularly valued for their exceptional nutritional profile, containing substantial amounts of proteins (26.62%), essential amino acids, vitamins, minerals, and bioactive compounds such as flavonoids (quercetin, kaempferol), phenolic acids (chlorogenic acid, gallic acid), and glucosinolates, which exhibit antimicrobial, antioxidant, and immunomodulatory properties [32, 37]. Water-soluble compounds in* Moringa* leaves, particularly benzyl isothiocyanate derived from glucosinolates, have demonstrated potent antimicrobial effects against aquatic borne pathogens, making them a promising candidate for water supplementation in aquaculture systems [56]. Additionally,* Moringa *seeds contain cationic proteins and polyelectrolytes that function as natural coagulants, potentially improving water clarity and reducing pathogen loads [63]. These multifunctional properties suggest that *Moringa *supplements could simultaneously enhance water quality parameters while delivering immunostimulatory compounds directly to fish through their gill surfaces and mucous membranes [61].

In contrast, the seeds, while beneficial for their natural coagulation properties and water purification potential [9], pose significant risks at high concentrations due to anti-nutritional factors and toxicity [38, 42]. Studies have shown that excessive use of *M. oleifera* seed powder can lead to fish mortality, with reported 96 h LC50 values of 124.0 mg/L in *Cyprinus carpio *[38]. This toxicity is attributed to compounds such as glucosinolates, phytates, saponins, and tannins, which can disrupt fish physiology and metabolism [48]. Exposure to seed extracts has been linked to hematological alterations, including reduced red blood cell counts, hemoglobin levels, and hematocrit values, indicating potential anemic conditions [1]. Furthermore, high concentrations of seed extracts can induce stress responses, alter enzyme activities, and inhibit growth rates [48].

The immune system of teleost fish relies heavily on cytokine-mediated responses to combat pathogens [36]. Pro-inflammatory cytokines such as Tumor Necrosis Factor-alpha (*TNF-α*), Interleukin- 1 beta (*IL- 1β*), and Interleukin- 6 (*IL- 6*) play pivotal roles in regulating inflammatory responses and enhancing disease resistance [64]. Dietary interventions using* M. oleifera *leaves offer promising opportunities to modulate these immune responses while avoiding the risks associated with seed supplements. Oxidative stress, a common challenge in intensive aquaculture systems, arises from an imbalance between reactive oxygen species (ROS) production and antioxidant defenses [16]. Key oxidative stress markers, including Superoxide Dismutase (SOD) activity and Malondialdehyde (MDA) levels, provide valuable insights into fish health and the efficacy of dietary interventions [66]. The natural antioxidants in* M. oleifera *leaves may help maintain oxidative homeostasis under intensive farming conditions [44].

One of the most pressing challenges in freshwater aquaculture is the threat posed by *Aeromonas hydrophila*, a highly destructive bacterial pathogen responsible for Motile Aeromonad Septicemia (MAS), which can cause mortality rates exceeding 80% during severe outbreaks [40, 58, 59]. The pathogen's ubiquity and increasing antibiotic resistance underscore the urgent need for effective prophylactic strategies that reduce reliance on antimicrobials [3, 60]. The intensification of catfish farming has highlighted the need for sustainable water supplements that enhance fish health performance [22]. Conventional water quality management often relies on chemical treatments that may accumulate in tissues, promote resistant pathogens, or cause environmental degradation [43]. This has spurred interest in plant-derived bioactive compounds that improve water quality, boost immunity, and protect against pathogens without harmful residues [6].

Despite the growing body of research on natural feed additives in aquaculture, significant knowledge gaps remain. While most studies have focused on *M. oleifera*as a dietary supplement [19, 45], limited research has explored its application as a water supplement, particularly the comparative effects of leaves versus seeds [18, 38]. Studies by Abouzied et al. [7] demonstrated improved water quality with *M. oleifera* extracts, while Rapatsa and Moyo [53] found that higher dosages of seed powder induced adverse effects, including histological alterations, hematological disruptions, and reduced growth in tilapia. Comprehensive studies examining the simultaneous effects of* M. oleifera* leaves and seeds on physiological, immunological, and disease-resistance parameters in African catfish are scarce, and the mechanisms underlying their modulation of immune responses and oxidative status remain poorly understood.

This study aimed to address these gaps by evaluating and comparing the effects of aqueous supplementation with *M. oleifera* leaf and seed powder on physiological parameters (liver and kidney functions), hematological indices (WBCs, PCV), stress indicators (glucose, cortisol), immune responses (*TNF-α*, *IL- 1β*, IL- 6),antioxidant/oxidative stress markers (SOD, MDA), and histopathological changes in African catfish. Additionally, the study investigated the potential of leaf supplementation to enhance resistance against *A. hydrophila* infection, providing insights into natural strategies for sustainable disease management in aquaculture.

## Materials and methods

### Ethical statement

The experiment was conducted at the Physiology Laboratory, Faculty of Veterinary Medicine, Cairo University. All experimental procedures were approved by the Institutional Animal Care and Use Committee (VET-CU-IACUC- 13102024982) and adhered to international guidelines for the care and use of laboratory animals.

### Moringa oleifera processing

*M. oleifera *leaf and seed powders were sourced from the Scientific Society of Moringa (National Research Centre, Dokki, Giza, Egypt). The leaves were washed with deionized water, air-dried under controlled conditions (25 ± 2 °C), and ground using an electric grinder (Moulinex, Grenoble, France). The resulting powder was sieved (60-mesh) to ensure uniform particle size [47]. On the other hand, moringa seeds were sorted, dehulled, and oven-dried at 50 °C for 10 h, following the method of Ijarotimi et al. [35] with minor modifications for aquafeed applications. The dried seeds were ground and sieved in the same manner as the leaves. Both powders were then stored in airtight containers and were sealed with aluminum foil until required.

### Experimental fish management

Adult African catfish (*C. gariepinus*) were purchased from a private fish farm in Kafr El Sheikh Governorate, Egypt, with an initial average body weight of 85.67 ± 3.2 g and length of 24.33 ± 0.5 cm. The fish were transported alive to laboratory facilities in oxygenated plastic bags in dark containers with minimal stress. Fish were acclimated for two weeks in glass aquarium (60 × 30 × 30 cm, 45 L capacity), equipped with continuous aeration and filled with dechlorinated tap water, and maintained under controlled conditions: temperature (24–26 °C), dissolved oxygen (6.5 ± 0.5 mg/L), and pH (7.2 ± 0.2). A 12:12 h light: dark photo period was maintained, and water quality was preserved by performing 40% water exchanges every other day.

### Experimental design

After acclimation, 135 fish were randomly assigned to three groups (GP1, GP2, GP3), with three replicates (15 fish per aquarium). All experimental groups received a basal diet; GP1 served as the control group, while GP2 and GP3 were additionally water supplemented with 0.8 g/L of *M. oleifera* leaf and seed powders, respectively, added after each water exchange. The selected concentration of 0.8 g/L was based on previous optimization studies demonstrating maximum efficacy for water purification [18]. Throughout the six-week experimental period, the experimental fish were fed a commercial diet (28% crude protein, Skretting, El Sharqia Governorate, Egypt) twice daily at 3% of their body weight, with feed amounts adjusted based on biomass. The uneaten feed was removed for one-hour post-feeding. Fish behavior and health were monitored daily, and water quality parameters were recorded throughout the study.

### Blood sample collection

At the end of the six-week study, fish were fasted for 24 h before blood sample collection. From each experimental group, five fish were randomly collected from each replicate aquarium (total *n* = 15 per treatment group) for analysis. For biochemical and hematological analyses, five fish from each treatment group were used, with each fish representing one biological replicate. Fish were anesthetized using benzocaine solution (100 mg/L) before blood collection. Blood samples were drawn from the caudal vein using sterile syringes. The blood was then divided into two portions for different analyses. For hematological analysis, blood was collected into EDTA tubes. For serum biochemical analysis, blood was collected in tubes without anticoagulants. These samples were centrifuged at 3000 rpm for 10 min at 4 °C, and the serum was stored at − 80°C until further analysis [26].

## Hematological and biochemical analysis

Hematological parameters, including PCV and WBC counts, were measured using standard methods for fish [28]. For biochemical analysis, commercial kits (Bio Diagnostic Co., Egypt) were used along with a Jasco-V530 UV–VIS spectrophotometer (JASCO, Tokyo, Japan). Liver function was assessed by measuring alanine aminotransferase (ALT) and aspartate aminotransferase (AST) activities, as described by Reitman and Frankel [54]. Kidney function was evaluated by measuring creatinine using the Jaffe reaction [33] and urea using the urease-GLDH enzymatic UV test [52]. Stress indicators, including serum glucose and cortisol levels, were measured using the GOD-PAP method [65] and a colorimetric assay [57], respectively.

## Oxidant/antioxidants analysis

Antioxidant enzyme analysis was conducted by measuring superoxide dismutase (SOD) activity using the phenazine methosulphate-mediated reduction of nitro blue tetrazolium dye at 560 nm [50]. The oxidative stress biomarker, lipid peroxidation, was evaluated by measuring malondialdehyde (MDA) concentrations using thiobarbituric acid reactive substances (TBARS) method with absorbance measured at 532 nm [51].

### Proinflammatory cytokine analysis

Proinflammatory cytokines (*TNF-α*, *IL- 1β*, IL- 6) in the serum were quantified using ELISA kits (Bio Diagnostic Co., Egypt) and expressed in picograms per milliliter (pg/mL), as described by Hanington and Belosevic [31].

### Histological analysis

Fish were euthanized using benzocaine solution at a concentration of 300 mg/L for 10 min, followed by decapitation to ensure death, following the guidelines of the American Veterinary Medical Association [11]. We collected tissue samples from the head kidney, spleen, liver, intestine, gills, and accessory respiratory organ through careful dissection. The collected tissues were processed in two ways: one portion was preserved in 10% neutral buffered formalin for histological examination following standard protocols [14], while the other portion was flash-frozen in liquid nitrogen and maintained at− 80 °C for subsequent gene expression analysis**.**

### Gene expression analysis

Total RNA was extracted from 100 mg of kidney and spleen tissues using the GeneJET RNA purification kit (Fermentas, UK), and RNA quality was verified via Nanodrop spectrophotometry (NanoDrop 2000, Thermo Scientific, Waltham, MA, USA) and agarose gel electrophoresis (1.5% agarose, Bio-Rad Laboratories, Hercules, CA, USA). First-strand cDNA synthesis was performed using Superscript II reverse transcriptase (Invitrogen) following the manufacturer's protocol. Briefly, 2 μg of total RNA was mixed with 1 μL of oligo(dT)20 primers (50 μM) and 1 μL of dNTP mix (10 mM each), brought to a volume of 13 μL with nuclease-free water, and incubated at 65 °C for 5 min. After cooling on ice, 4 μL of 5 × First-Strand Buffer, 1 μL of DTT (0.1 M), 1 μL of RNaseOUT, and 1 μL of Superscript II RT were added. The reaction was incubated at 42 °C for 50 min, followed by inactivation at 70 °C for 15 min. Quantitative real-time PCR (qPCR) was performed using a Step One Plus Real-Time PCR System (Applied Biosystems) with SYBR Green PCR Master Mix (Thermo Scientific, MA, USA). Each reaction mixture (20 μL total volume) contained 10 μL SYBR Green Master Mix, 0.6 μL each of forward and reverse primers (10 μM) for the target genes (*IL- 1β* and *TNF-α*) and the reference gene (*β-actin*), as listed in Table 1, μL cDNA template, and nuclease-free water. The thermal cycling conditions were programmed as follows: initial denaturation at 94 °C for 10 min, followed by 40 cycles of denaturation at 94 °C for 30 s, annealing at 55 °C for 45 s, and extension at 74 °C for 10 s. A melting curve analysis (65–95 °C with 0.5 °C increments) was performed to confirm amplification specificity. The relative expression levels of the target genes (*IL- 1β* and *TNF-α*) were normalized to the reference gene (*β-actin*) and calculated using the 2-ΔΔCt method [41], with results expressed as fold changes relative to the control group**.**

**Table 1:** Primers used for gene expression analysis

Target gene	Primer sequence (5'− 3')	Amplicon size (bp)	GenBank Accession No	Reference
*IL- 1β*	F: TGCAGTGAATCCAAGAGCTACAGC R: CCACCTTTCAGAGTGAATGCCAGC	128	MH341527.1	(Nasrullah et al. [49])
*TNF-α*	F: TCTCAGGTCAATACAACCCGC R: GAGGCCTTTGCGGAAAATCTTG	125	XM_053490102.1	(Abdel Rahman et al. [4])
*β-actin*	F: ACCGGAGTCCATCACAATACCAGT R: GAGCTGCGTGTTGCCCCTGAG	192	JN806115.1	(Nasrullah et al. [49])

### Experimental challenge with *A. hydrophila*

After 6 weeks of aqueous exposure to *M. oleifera* powders, all fish were fasted for 24 h before challenge initiation. A virulent strain of *A. hydrophila* (T11201), obtained from the Department of Aquatic Animal Medicine, Faculty of Veterinary Medicine, Cairo University, was used for the challenge study. The strain was molecularly characterized by *gyrB* gene sequencing (OL321922.1) and confirmed positive for virulence genes including hemolysin (*hlyA*), aerolysin (*aer*), lipase (*lip*), and cytotonic heat-stable enterotoxin (*ast*) [21]. For challenge preparation, bacterial cultures were grown on tryptic soy agar for 24 h at 28 °C, then harvested and suspended in sterile physiological saline (0.85% NaCl). The bacterial suspension was adjusted spectrophotometrically at 600 nm wavelength and confirmed by plate counting to achieve a final concentration of 1.0 × 10^6^CFU/mL, representing a sub-lethal dose based on preliminary LD50 trials [5]. For the challenge experiment, 60 fish in total were used (20 fish per treatment group, in three replicate tanks). The experimental design included both a negative control (non-infected fish) and a positive control (infected fish without prior Moringa treatment). Fish in each group were divided as follows: (1) Control group: 10 fish received sterile saline (negative control) and 10 fish received bacterial suspension (positive control); (2) Leaves-treated group (GP2): all 20 fish received bacterial suspension; and (3) Seeds-treated group (GP3): all 20 fish received bacterial suspension. All injections were administered intraperitoneally at a volume of 0.2 mL. Clinical signs, behavior, and mortality were monitored for 14 days post-challenge. Pathogen-specific mortality was confirmed via bacterial re-isolation, and cumulative survival rates were calculated.

### Statistical analysis

Data was analyzed using SPSS version 21 (IBM Corp., Armonk, USA). Normality was verified using Shapiro–Wilk’s test. One-way ANOVA with Duncan’s multiple range test compared treatment effects. Results were expressed as mean ± standard error (SE), with significance set at *P* ≤ 0.05.

## Results

### Fish weight and clinical observations

**Fig. 1:**
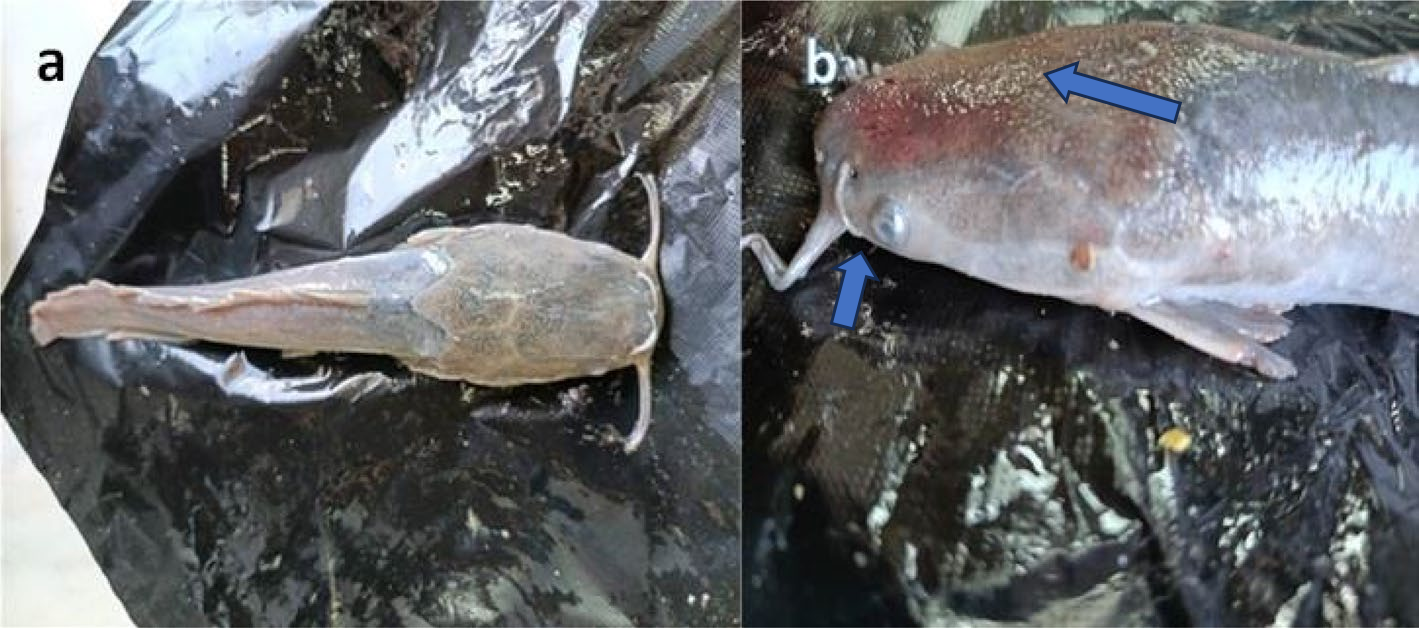
Clinical signs observed in African catfish (*C. gariepinus*) after 6 weeks of exposure to *M. oleifera* seeds treatment: (**a**) body showing growth retardation and skin discoloration with patchy depigmentation (long arrow), and (**b**) head region exhibiting exophthalmia (short arrow) and hyperemia

Moringa leaves enhanced growth performance while seeds reduced growth and induced adverse clinical signs in treated catfish. Final weight was monitored over six weeks in the three treatment groups (Table 2). Starting from similar initial weights (80.0–81.0 g), both control and Moringa leaves groups showed significant growth (*P* ≤ 0.05), reaching 109.0 ± 5.4 g and 120.5 ± 0.7 g, respectively, while the Moringa seeds group showed reduced growth (91.5 ± 17.2 g). Clinical observations remained normal in both control and leaves-treated groups, while the seeds group exhibited adverse effects including reduced appetite, skin discoloration, hyperemia, and exophthalmia (Fig. 1).

**Table 2:** Growth performance and clinical observations in african catfish during 6-Week treatment period

Parameter	Control	Moringa leaves	Moringa seeds
**Growth performance**			
Initial weight (gm)	80.0 ± 13.5	79.0 ± 8.9	81.0 ± 11.2
Final weight (gm)	109.0 ± 5.4*	120.5 ± 0.7*	90.5 ± 17.2
**Clinical signs**			
Appetite	Normal	Normal	Reduced
Skin	Normal	Normal	Skin discolouration, and hyperaemia
Eye condition	Normal	Normal	Exophthalmia

Values represent mean ± SE. Asterisk (*) indicates significant difference from initial weight (*P* ≤ 0.05)

### Hematological and biochemical responses

Both Moringa treatments increased white blood cell counts, with seeds causing significant elevations in liver and kidney function markers and stress indicators, while leaves produced minimal changes.

Hematological analysis revealed differential responses among treatment groups (Table 3). PCV values showed no differences between treated groups compared to control (*P* ≤ 0.05), while WBC counts increased significantly (*P* ≤ 0.05) in both Moringa-treated groups compared to the control (9.53 ± 1.03 × 10^3^ cells/mm^3^), with the seeds group showing the highest elevation (11.07 ± 1.23 × 10^3^ cells/mm^3^), followed by the leaves group (10.63 ± 0.29 × 10^3^ cells/mm^3^).

**Table 3:** Physiological Parameters in African Catfish After 6 Weeks of Moringa Treatments

Parameter	Control	Moringa Leaves	Moringa Seeds
**Haematological parameters**			
PCV (%)	29.33 ± 1.25	29.33 ± 1.70	29.00 ± 0.82
WBCs (cells/mm^3^)	9.53 ± 1.03 × 10^3^	10.63 ± 0.29 × 10^3^*	11.07 ± 1.23 × 10^3^*
**Liver function**			
ALT (U/L)	17.8 ± 0.3	18.2 ± 0.6	30.2 ± 1.1*
AST (U/L)	34.6 ± 0.7	35.8 ± 1.1	53.0 ± 1.6*
**Kidney function**			
Creatinine (mg/dL)	0.35 ± 0.01	0.38 ± 0.02	0.69 ± 0.04*
Urea (mmol/L)	23.0 ± 0.4	24.2 ± 0.7	39.8 ± 0.9*
**Stress indicators**			
Glucose (mg/dL)	74.8 ± 2.6	76.9 ± 3.4	113.3 ± 3.8*
Cortisol (μg/dL)	14.1 ± 0.8	15.3 ± 1.0	27.4 ± 1.3*
**Oxidative status**			
SOD (U/mL)	11.7 ± 0.4	15.1 ± 0.5*	7.4 ± 0.6*
MDA (nmol/mL)	16.2 ± 0.5	12.1 ± 0.4*	27.2 ± 1.0*
**Serum cytokines**			
* TNF-α* (pg/mL)	29.0 ± 0.5	33.2 ± 0.5*	44.3 ± 1.0*
*IL- 1β* (pg/mL)	11.2 ± 0.4	14.1 ± 0.4*	19.6 ± 0.4*
IL- 6 (pg/mL)	52.3 ± 0.6	58.8 ± 0.5*	68.3 ± 1.1*

Values represent mean ± SE (*n* = 5 for biochemical parameters)

Asterisk (*) indicates significant difference from control group (*P* ≤ 0.05)

Significant alterations were observed in liver function markers in the seeds group, with ALT and AST levels significantly higher (*P* ≤ 0.05) compared to control, while leaves treatment resulted in slight elevations (Table 3). Similarly, kidney function markers (creatinine and urea) and stress indicators (glucose and cortisol) were significantly increased in the seeds group (*P* ≤ 0.05), while the leaves group showed minor changes compared to control values (Table 3).

Stress indicator analysis revealed a similar pattern, with the seeds group exhibiting significant increases (*P* ≤ 0.05) in both glucose and cortisol levels compared to control values, while the leaves group-maintained stress indicators near control levels in both parameters (Table 3).

#### Oxidant/antioxidant response

Moringa treatments produced opposite effects on oxidative status, with leaves enhancing antioxidant activity while seeds compromised oxidative defenses. Oxidative**/** antioxidant parameters showed significant changes in both treatment groups but in opposite directions (Table 3). SOD activity increased significantly (*P* ≤ 0.05) in the leaves group but decreased significantly in the seeds group compared to control. MDA levels showed a similar pattern of significant changes (*P* ≤ 0.05), with marked elevation in the seeds group and significant reduction in the leaves group compared to control.

#### Serum cytokine response

Both Moringa treatments upregulated pro-inflammatory cytokines, with seeds inducing more pronounced inflammatory responses than leaves. Cytokines showed significant changes (*P* ≤ *0.05*) in both treatment groups, with more pronounced elevations in the seeds group (Table 3). *TNF-α*, *IL- 1β*, and *IL- 6* levels were significantly increased in both Moringa-treated groups compared to their level in control group, with the seeds group consistently showing higher cytokine elevations than the leaves group. This pattern of differential cytokine upregulation suggests a stronger inflammatory response in seed-treated fish compared to leaf-treated fish.

#### Gene expression analysis of IL- 1β and TNF-α

RT-PCR revealed significant upregulation of pro-inflammatory cytokine genes in both treatment groups, with seeds inducing higher expression levels than leaves across three time points (2, 4, and 6 weeks).

**Fig. 2:**
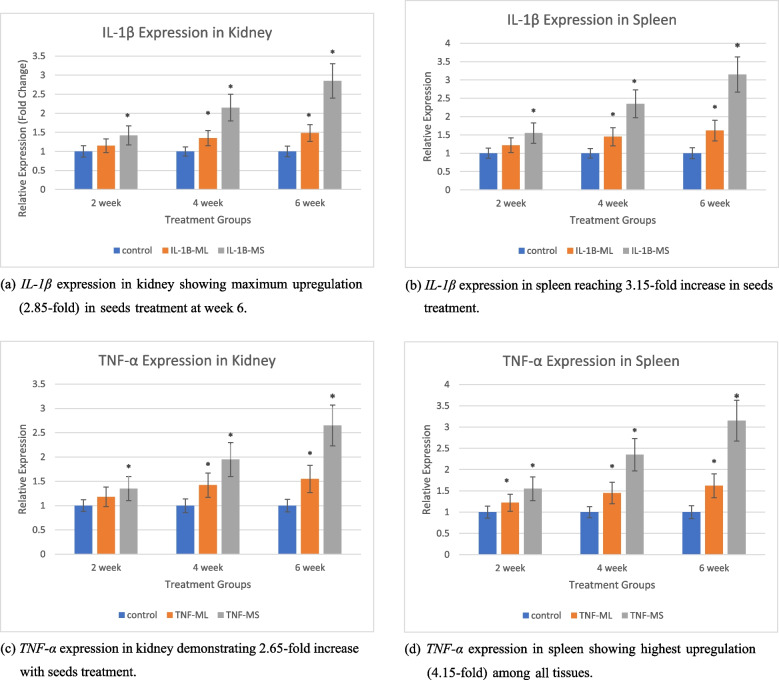
Expression levels of pro-inflammatory cytokines *IL- 1β* and *TNF-α* mRNA in kidney and spleen tissues of *C. gariepinus* exposed to *M. oleifera* treatments over 6 weeks. Asterisks indicate significant differences from control group (*P ≤ 0.05). **a** *IL- 1β* expression in kidney showing maximum upregulation (2.85-fold) in seeds treatment at week 6. **b** *IL- 1β* expression in spleen reaching 3.15-fold increase in seeds treatment. **c** *TNF-α* expression in kidney demonstrating 2.65-fold increase with seeds treatment. **d** *TNF-α* expression in spleen showing highest upregulation (4.15-fold) among all tissues

RT-PCR analysis revealed significant upregulation of *IL- 1β* and *TNF-α* in kidney and spleen tissues across both treatment groups, with more pronounced effects in the seeds group (Fig. 2). Gene expression was analyzed at three points (2, 4, and 6 weeks) to monitor the progression of immunomodulatory effects. In kidney tissue at week 6, *IL- 1β* expression showed significant elevation (*P* ≤ 0.05) in both seeds (2.85-fold) and leaves (1.48-fold) groups, with the seeds group showing more pronounced elevation. Similarly, *TNF-α* expression patterns showed significant upregulation with greatest effects observed at week 6, where seeds treatment induced 2.65-fold increase in kidney and 4.15-fold increase in spleen, compared to moderate increases in the leaves group (1.55-fold in kidney and 2.48-fold in spleen).

#### Histological findings

**Fig. 3:**
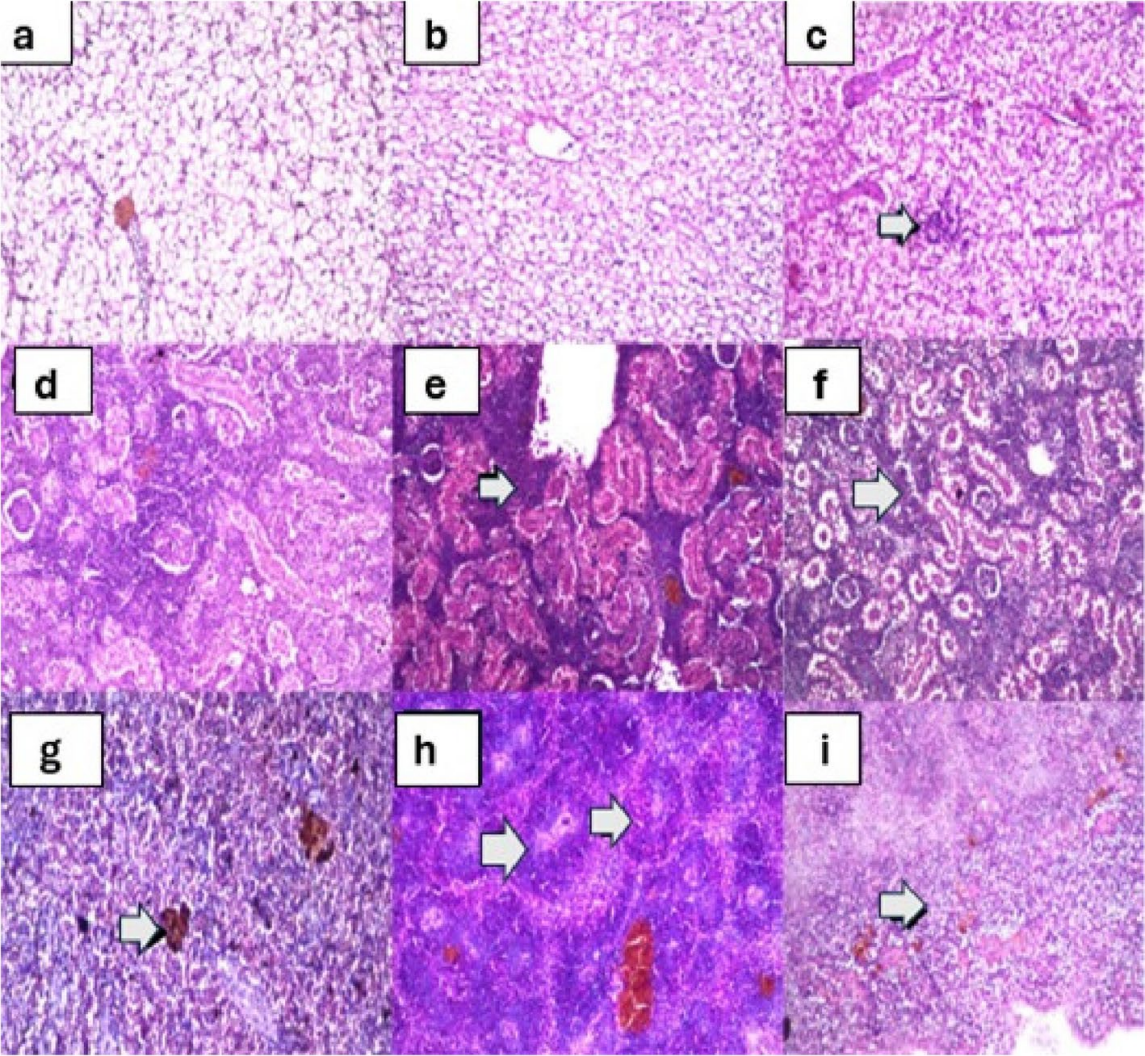
Comparative histological analysis of liver, kidney, and spleen tissues from African catfish (*C. gariepinus*) after 6-week exposure to Moringa treatments (H&E staining). Liver sections showing: (**a**) normal histological structure with intact central vein and hepatocytes in control group, (**b**) normal liver architecture in leaves-treated group, and (**c**) focal lymphoid aggregation in parenchyma (arrow) in seeds-treated group. Kidney sections showing: (**d**) normal glomeruli and tubules in control group, (**e**) massive lymphoid aggregation (arrow) and focal hemorrhages in leaves-treated group, and (**f**) lymphoid hyperplastic proliferation between tubules and glomeruli in seeds-treated group. Spleen sections showing: (**g**) normal lymphoid structure with melanin pigmentation (arrow) in control group, (**h**) lymphoid proliferation in follicles (arrow) in leaves-treated group, and (**i**) diffuse lymphoid proliferation (arrow) in follicles in seeds-treated group. Hematoxylin and eosin stain (X 200)

**Fig. 4:**
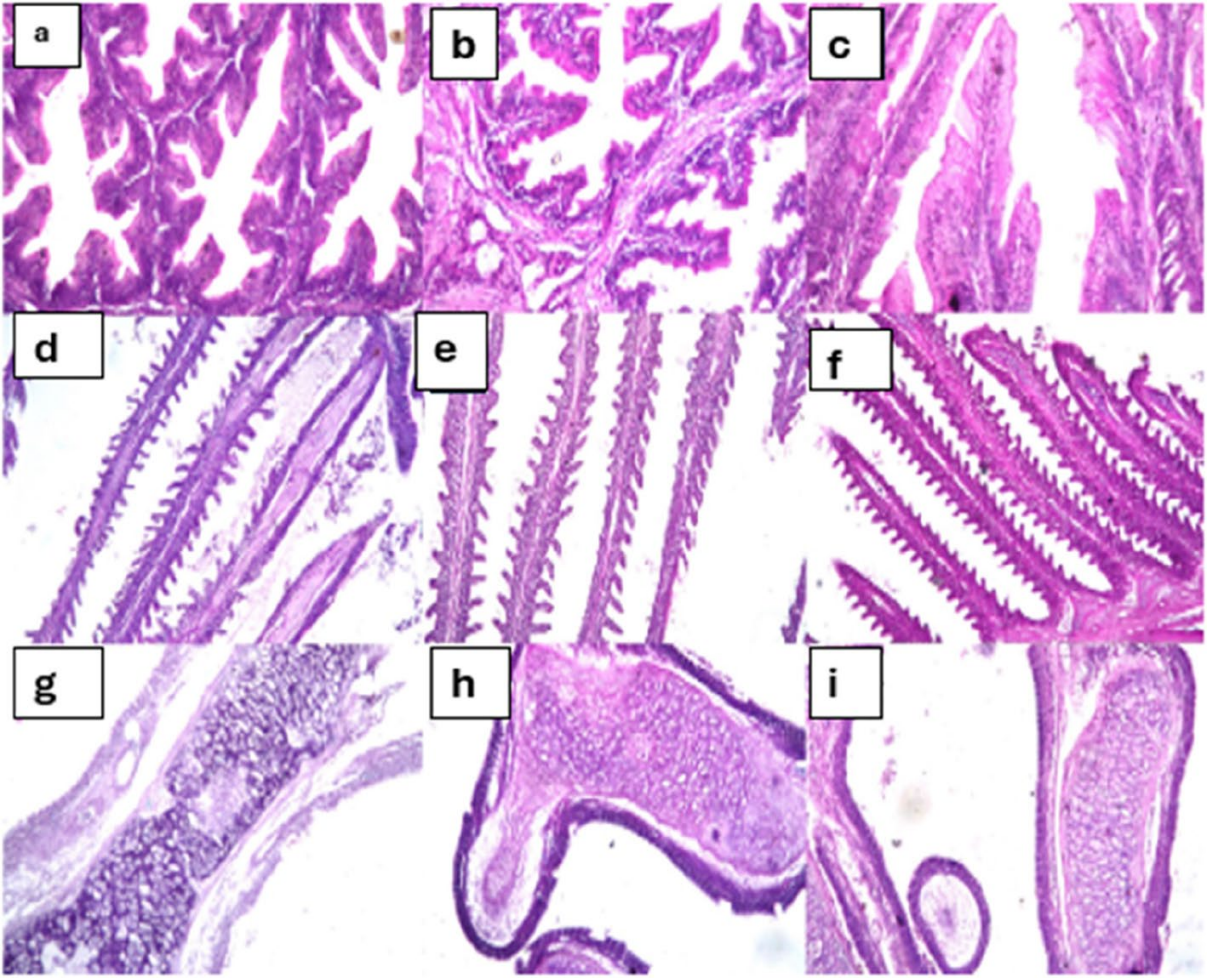
Comparative histological analysis of intestine, gills, and accessory respiratory organ of African catfish (*C. gariepinus*) after 6-week exposure to Moringa treatments (H&E staining). Intestinal sections showing: (**a**) normal mucosal layer with intact epithelium and lamina propria of villi in the control group, (**b**) normal intestinal architecture in leaves-treated group, and (**c**) preserved intestinal structure in seeds-treated group (X 200). Gill sections showing: (**d**) normal filaments and lamellae structure in the control group, (**e**) normal gill architecture in leaves-treated group, and (**f**) preserved gill organization in seeds-treated group (X100). Accessory respiratory organ sections showing: (**g**) normal parenchymal and stromal structure in the control group, (**h**) normal architecture in leaves-treated group, and (**i**) preserved tissue organization in seeds-treated group (X200)

Histological examination revealed treatment-specific tissue responses, with both Moringa treatments inducing immunological activation while seeds caused more pronounced changes in immune-related organs. Histological examination revealed distinct tissue responses across treatment groups (Figs. 3 and 4). In liver sections, control fish (Fig. 3a, H&E, × 200) displayed normal hepatic architecture with well-defined hepatocytes arranged in cords around central veins. The leaves-treated group maintained similar normal architecture (Fig. 3b, H&E, × 200) with intact hepatocytes and sinusoids. In contrast, the seeds-treated group (Fig. 3c, H&E, × 200) exhibited notable alterations including focal lymphoid aggregation, mild vacuolation of hepatocytes, and occasional pyknotic nuclei indicating early degenerative changes.

Kidney sections from control fish (Fig. 3d, H&E, × 200) showed normal nephrons with well-defined glomeruli and tubules. The leaves-treated group (Fig. 3e, H&E, × 200) demonstrated significant immune cell infiltration with massive lymphoid aggregation between glomeruli and tubules and focal haemorrhages, indicating active immune responses without structural damage. Seeds-treated fish (Fig. 3f, H&E, × 200) displayed more pronounced changes, including lymphoid hyperplastic proliferation, tubular epithelial degeneration, and increased Melano macrophage centres, suggesting both immune activation and mild nephrotoxicity. Splenic tissue from control fish (Fig. 3g, H&E, × 200) exhibited normal white and red pulp organization with characteristic melanin pigmentation. Leaves-treated fish (Fig. 3h, H&E, × 200) showed marked lymphoid proliferation in follicles with enlarged white pulp areas, indicating immunostimulant. Seeds-treated spleen (Fig. 3i, H&E, × 200) demonstrated diffuse lymphoid proliferation, hemosiderin deposition, and expanded Melano macrophage centers, suggesting more intense immune activation with possible haemolytic effects.

In contrast, digestive and respiratory organs maintained normal architecture across all treatment groups (Fig. 4). The intestinal tissue showed normal histological structure of the mucosal layer with intact lining epithelium and underlying lamina propria of the villi in control (Fig. 4a, H&E, × 200), leaves-treated (Fig. 4b, H&E, × 200), and seeds-treated groups (Fig. 4c, H&E, × 200). Similarly, gill tissue displayed normal histological organization of filaments and lamellae across control (Fig. 4d, H&E, × 100), leaves-treated (Fig. 4e, H&E, × 100), and seeds-treated groups (Fig. 4f, H&E, × 100). The accessory respiratory organ maintained normal parenchymal and stromal structure in control (Fig. 4g, H&E, × 200), leaves-treated (Fig. 4h, H&E, × 200), and seeds-treated fish (Fig. 4i, H&E, × 200), with no treatment-related alterations observed.

## Challenge test with *A. hydrophila*

**Fig. 5:**
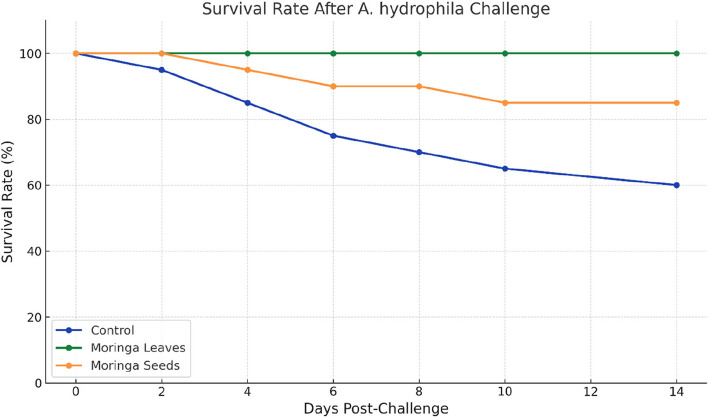
Survival rates of *C. gariepinus* challenged with *A. hydrophila* (1.0 × 10^6^ CFU/mL) after six weeks of exposure to different treatments. Control group showed progressive mortality reaching 40% by day fourteen, while Moringa leaves-treated group maintained 100% survival throughout the observation period. The Moringa seeds-treated group showed intermediate protection with 85% survival. Fish monitored for 14 days post-challenge

Moringa leaves provided complete protection against *A. hydrophila* challenge (100% survival), while seeds offered intermediate protection (85% survival) compared to control fish (60% survival). Following the six-week exposure period, fish were challenged with *A. hydrophila* to evaluate disease resistance (Fig. 5). Clinical signs appeared within 24 h post-challenge, with fish exhibiting reduced feeding activity and erratic swimming behaviour. The control group showed the most severe clinical manifestations, including haemorrhages, skin hyperemia, epidermal erosion, and ulcerative lesions. Mortality in the control group began 48 h post-challenge and progressively increased, reaching a cumulative mortality rate of 40% by day fourteen, with survival rate declining to 60% by the end of the observation period.

Fish exposed to Moringa leaves demonstrated superior disease resistance, maintaining a significantly higher survival rate (*P* ≤ 0.01) compared to both control and seeds groups, with 100% survival throughout the 14-day post-challenge period. These fish exhibited minimal clinical signs, primarily limited to temporary reduction in feeding activity during the first 72 h post-challenge. This enhanced disease resistance correlates with the observed immunostimulatory effects, including elevated WBC counts, balanced pro-inflammatory cytokine expression, and enhanced antioxidant status in the leaves-treated group.

The Moringa seeds group showed intermediate resistance to *A. hydrophila* infection, maintaining 85% survival by day fourteen. Clinical signs in this group were moderate, including scattered haemorrhagic spots and mild fin erosion. While the severity of symptoms was significantly lower compared to the control group, the reduced survival rate compared to the leaves group may be attributed to the physiological stress and compromised antioxidant status observed in these fish. Bacterial re-isolation confirmed *A. hydrophila* as the cause of mortality, with the highest bacterial loads recovered from control group tissues, while significantly lower counts are observed in surviving fish from both Moringa treatment groups (*P* < 0.05).

## Discussion

This study provides comprehensive insights into the physiological and immunological effects of *M. oleifera* leaves and seeds supplements on African catfish (*C. gariepinus*), revealed a complex interplay between immune activation and potential toxicity. The novel approach of water supplementation, as opposed to traditional dietary inclusion methods [19, 22], has unveiled distinct response patterns in immune stimulation and organ function that warrant careful consideration for aquaculture applications. The findings demonstrate significant differences between leaves and seeds treatments, with leaves promoting beneficial effects, while seeds exhibited potential toxicological concerns at the tested concentrations.

The growth performance results revealed a significant impact of *M. oleifera*supplementation. While the control group showed steady growth (109.0 ± 5.4 g), the leaves treated group demonstrated the highest growth performance (120.5 ± 0.7 g), suggesting potential growth-promoting effects. This improvement may be attributed to the exceptional nutritional composition of Moringa leaves, which contain 23–30% crude protein, 5.9% crude fiber, 7.6–12% ash, and 7.09% crude lipid in their dried form, providing essential macronutrients necessary for tissue development and metabolic functions [37, 55, 62]. In contrast, the seeds treated showed reduced growth (90.5 ± 17.2 g) and clinical abnormalities, aligning with previous studies reporting growth inhibition in fish exposed to high concentrations of* Moringa* seeds extracts [1, 12]. This growth suppression may be linked to the presence of antinutritional factors in* Moringa* seeds, such as glucosinolates, phytates, and tannins, which can interfere with nutrient absorption and metabolic processes [48]. These compounds may inhibit digestive enzymes or bind essential nutrients, reducing their bioavailability and leading to impaired growth [46].

The hematological analysis revealed notable variations in immune response parameters across treatment groups. The marked increase in white blood cell (WBC) counts, particularly in the seeds treated group (11.07 ± 1.23 × 10^3^ cells/mm.^3^), suggests a strong immunostimulatory effect, consistent with the findings that *Moringa *can modulate immune responses [10, 44]. However, such immune activation may come at a physiological cost, especially in the seed-treated group. While elevated WBC counts in both treatment groups reflect enhanced immune responses [36], the more pronounced elevation in the seeds group, coupled with increased stress markers, suggests a stress-induced immune response rather than beneficial immunostimulant. This could indicate the intricate relationship between stress and the immune system and ensure that the immune system is being overactivated, potentially leading to chronic immune exhaustion, which is proud with the finding of [8].

The biochemical analysis revealed significant hepatic and renal responses to *Moringa* treatments. While the leaves group showed non-significant slight elevations in liver enzymes, the seeds group demonstrated marked increases in ALT (30.2 ± 1.1 U/L) and AST (53.0 ± 1.6 U/L) compared to the control, suggesting potential hepatocellular injury [23, 38]. Similarly, kidney function markers showed only slight increases in the leaves group but significant elevation in the seeds group. These findings align with Abbas & El-Badawi [1], who reported similar hepatic dysfunction in fish exposed to high concentrations of *Moringa* seeds extracts. In contrast, the minimal elevation of these enzymes in the leaves group may indicate a more balanced physiological response, related to enhanced metabolic activity rather than tissue damage.

The stress response analysis provided crucial insights into the physiological impact of *Moringa *supplementation. The seeds group showed significant increases in both glucose (113.3 ± 3.8 mg/dL) and cortisol (27.4 ± 1.3 μg/dL), while the leaves group exhibited only slight, non-significant elevations (glucose: 76.9 ± 3.4 mg/dL, cortisol: 15.3 ± 1.0 μg/dL), suggesting a more balanced physiological response [67]. The minimal increases observed in the leaves group might represent an adaptive response rather than chronic stress, as supported by the better survival rates and disease resistance in this group.

On the other hand, the oxidative stress parameters revealed contrasting effects between leaves and seeds treatments. *Moringa* leaves supplementation resulted in enhanced antioxidant defense, as evidenced by increased superoxide dismutase (SOD) activity (15.1 ± 0.5 U/mL), consistent with other studies showing the antioxidant properties of *Moringa* [20, 36]. The lower SOD levels in seeds treated fish (7.4 ± 0.6 U/mL) and the accompanying increase in lipid peroxidation (MDA: 27.2 ± 1.0 nmol/mL) suggest that the seeds may compromise antioxidant defense, contributing to oxidative stress, possibly due to the pro-oxidant effects of certain seeds components at higher concentrations. This result supports the findings of [8, 39], who observed that excessive immune activation could impair antioxidant mechanisms, leading to oxidative damage. The hepatic and kidney stress responses, combined with oxidative damage (indicated by increased MDA levels), suggest that while Moringa seeds offer water purification benefits, their bioactive compounds may stress vital organs when used over extended periods [12, 38].

In seeds treated fish, the upregulation of pro-inflammatory cytokines, particularly *TNF-α* (4.15-fold in the spleen) and *IL- 1β* (3.15-fold in the spleen), underscores a potent immunostimulatory response. Elevated cytokine levels have shown to correlate with tissue damage and metabolic stress [13]. While indicating immune activation, this inflammatory response may contribute to physiological strain, as evidenced by the increased glucose and cortisol levels, which suggest metabolic disruption. The moderate increase in pro-inflammatory cytokines (*TNF-α*, *IL- 1β*, IL- 6) in the leaves group suggests enhanced immune preparedness without excessive inflammation, supporting the findings of [8, 64] regarding balanced cytokine responses in fish.

The histological findings provided structural evidence supporting biochemical and immunological observations. Histological analysis further supports this interpretation, with seeds treatment leading to extensive lymphoid proliferation in the spleen and kidney, indicating systemic immune activation beyond beneficial levels. Similar findings have been reported in studies using plant-based supplements, where immune stimulation could lead to unintended tissue stress [13]. The enhanced lymphoid proliferation in immunological tissues of leaves treated fish, without considerable damage to other organs, suggests beneficial immunomodulation. This agrees with Abdel-Latif et al. [3] observations regarding the immunostimulatory effects of moringa leaves.

Notably, the most significant finding was the enhanced disease resistance against *A. hydrophila *challenge in the leaf-treated group, demonstrated by a 100% survival rate and minimal clinical signs. This superior protection may be attributed to the combination of enhanced immune responses and maintained physiological homeostasis, supporting the potential of moringa leaves as a natural immunostimulant in aquaculture. This supports the growing body of literature highlighting the potential of Moringa as a natural disease resistance enhancer [20, 24]. The leaves treatment achieved this enhanced resistance without the accompanying physiological stress seen with seeds, making it a safer and more sustainable option for aquaculture applications.

The superior health outcomes observed in fish treated with *Moringa *leaves may be attributed to multiple physiological mechanisms. Enhanced antioxidant status, evidenced by increased SOD activity (15.1 ± 0.5 U/mL) and reduced lipid peroxidation (MDA: 12.1 ± 0.4 nmol/mL), likely results from the synergistic action of polyphenolic compounds including quercetin, kaempferol, and chlorogenic acid, which activate nuclear factor erythroid 2-related factor 2 (Nrf2) signaling pathways [34]. Moderate upregulation of pro-inflammatory cytokines (*TNF-α*: 2.48-fold, *IL- 1β*: 1.62-fold in spleen) observed in leaves treated fish suggests activation of the nuclear factor kappa B (NF-κB) pathway at levels sufficient to prime immune responses without inducing inflammatory damage [8, 68]. This balanced immune activation is mediated by isothiocyanates and other glucosinolate-derived compounds that modulate macrophage activity and cytokine production. This multi-target mechanism explains why leaf treatment conferred complete protection against* A. hydrophila* challenge while maintaining physiological homeostasis.

In conclusion, while Moringa seeds possess powerful immunostimulatory properties, their use as water supplements requires careful consideration due to the potential for toxicity. These findings concise with previous studies [12, 38], underscoring the need for a balanced approach when using natural supplements in aquaculture.* Moringa* leaves present a safer and more effective alternative, offering robust immune stimulation without significant organ stress, suggesting their potential as sustainable health promoters in aquaculture systems. Future studies should focus on optimizing application methods and concentrations for *Moringa* leaves while exploring improved extraction methods for seeds to maximize benefits and minimize toxicity. Additionally, understanding the molecular mechanisms underlying the differential effects of leaves and seeds could lead to the development of more effective and environmentally sustainable disease management strategies in aquaculture, contributing to the industry's sustainable growth.

## Data Availability

All data generated or analyzed during this study are included in this article.
